# Risk of Heart Failure Hospitalization Associated With Hypoxia‐Inducible Factor Prolyl Hydroxylase Inhibitors Versus Erythropoiesis‐Stimulating Agents: A Nationwide Claims‐Based Cohort Study

**DOI:** 10.1002/pds.70453

**Published:** 2026-08-29

**Authors:** Yuki Kunitsu, Keiko Ikuta, Daiki Hira, Shunsaku Nakagawa, Tomohiro Terada

**Affiliations:** ^1^ Department of Clinical Pharmacology and Therapeutics Kyoto University Hospital Kyoto Japan; ^2^ Graduate School of Pharmaceutical Sciences Kyoto University Kyoto Japan

**Keywords:** erythropoiesis‐stimulating agent, heart failure, hypoxia‐inducible factor prolyl hydroxylase inhibitor, pharmacoepidemiology, renal anemia

## Abstract

**Purpose:**

Hypoxia‐inducible factor prolyl hydroxylase inhibitors (HIF‐PHIs) are increasingly used to treat renal anemia in patients with chronic kidney disease (CKD). We evaluated the risk of heart failure associated with HIF‐PHIs compared with erythropoiesis‐stimulating agents (ESAs) in routine clinical practice.

**Methods:**

We conducted a nationwide, retrospective, active‐comparator cohort study using a Japanese claims database. We included adults with non‐dialysis CKD and heart failure initiating HIF‐PHIs or ESAs alongside diuretic therapy between August 2020 and March 2025. The primary outcome was heart failure hospitalization. Secondary outcomes included emergency heart failure hospitalization and hospitalization requiring intravenous diuretics. Baseline characteristics were balanced using inverse probability of treatment weighting (IPTW) based on propensity scores. Hazard ratios (HRs), 95% confidence intervals (CIs), and sensitivity analyses were evaluated in predefined subgroups using Cox proportional hazards models.

**Results:**

The cohorts comprised 14 995 HIF‐PHI and 22 889 ESA users with baseline characteristics well balanced after IPTW adjustment. Heart failure hospitalization incidence rates per 1000 person‐years (PY) were 157.8 for HIF‐PHIs and 177.0 for ESAs. HRs were 0.93 [95% CI 0.87–1.01] for heart failure hospitalization, 0.94 [95% CI 0.84–1.03] for emergency heart failure, and 0.87 [95% CI 0.81–0.94] for intravenous diuretic administration. Results were generally consistent across subgroup and sensitivity analyses.

**Conclusions:**

Although residual confounding cannot be excluded because of the observational study design, HIF‐PHIs were not associated with evidence of an increased risk of heart failure hospitalization in patients with heart failure.

## Key Points

- Concerns have been raised regarding the risk of worsening heart failure associated with hypoxia‐inducible factor prolyl hydroxylase inhibitors (HIF‐PHIs) among patients with heart failure.
- Evidence regarding heart failure outcomes associated with HIF‐PHIs in routine clinical practice remains limited.
- In this nationwide Japanese claims‐based cohort study, HIF‐PHIs were not associated with an increased risk of heart failure hospitalization compared with erythropoiesis‐stimulating agents (ESAs).
- Similar findings were observed for emergency heart failure hospitalization and hospitalization requiring intravenous diuretics.
- The results were consistent across subgroup and sensitivity analyses, providing complementary real‐world evidence regarding heart failure‐related outcomes associated with HIF‐PHIs among patients with heart failure.

## Plain Language Summary

Hypoxia‐inducible factor prolyl hydroxylase inhibitors (HIF‐PHIs) are newer medicines used to treat anemia in people with chronic kidney disease. However, concerns have been raised that these medicines might worsen heart failure, especially in patients with pre‐existing heart failure. To investigate this issue, we analyzed healthcare claims data from a large nationwide database in Japan. We compared patients with heart failure who started HIF‐PHIs with similar patients who started conventional anemia treatments called erythropoiesis‐stimulating agents (ESAs), evaluating hospitalizations for heart failure and related outcomes. Among more than 37 000 patients, we found that HIF‐PHIs were not associated with a higher risk of heart failure hospitalization compared with ESAs. Similar results were observed when examining emergency hospitalizations and other indicators of worsening heart failure, and additional analyses using different statistical approaches produced consistent findings. These results suggest that HIF‐PHIs do not appear to substantially increase the risk of worsening heart failure in routine clinical practice, providing important information for clinicians treating anemia in patients with chronic kidney disease and heart failure.

## 1 Introduction

Hypoxia‐inducible factor prolyl hydroxylase inhibitors (HIF‐PHIs) are a novel class of oral agents for the treatment of renal anemia. They promote endogenous erythropoietin production through the stabilization of hypoxia‐inducible factor (HIF) pathways while also improving iron metabolism [1, 2, 3]. HIF‐PHIs have become an important therapeutic option for renal anemia management in patients with chronic kidney disease (CKD), including those with non‐dialysis CKD.

Despite their therapeutic advantages, concerns have been raised regarding the cardiovascular safety of HIF‐PHIs, including thromboembolic events, fluid retention, and worsening heart failure [4, 5]. Experimental studies and clinical trials have further highlighted the potential cardiovascular effects of HIF pathway activation–specifically its impact on fluid homeostasis and heart failure physiology [6, 7, 8]—although the underlying mechanisms remain incompletely understood.

A recent secondary analysis of the ASCEND‐ND [4] and ASCEND‐D [9] randomized controlled trials evaluated heart failure outcomes among patients receiving daprodustat compared with erythropoiesis‐stimulating agents (ESAs) [7]. In non‐dialysis patients, daprodustat was associated with a higher risk of first heart failure hospitalization (hazard ratio [HR] 1.22; 95% confidence interval [CI]: 0.95–1.56), and this risk appeared greater among patients with a history of heart failure (HR 1.37; 95% CI: 0.89–2.11) [7]. Furthermore, daprodustat was significantly associated with total (first and recurrent) heart failure hospitalization events in non‐dialysis patients (HR 1.46; 95% CI: 1.11–1.92) [7]. Although statistically significant increases were not consistently observed across all outcomes, these findings raised concerns regarding the potential risk of worsening heart failure associated with HIF‐PHI therapy, particularly in high‐risk non‐dialysis CKD patients.

However, current evidence has several limitations. The ASCEND trials evaluated heart failure outcomes as secondary endpoints and excluded patients with severe heart failure or recent cardiovascular events [4, 9]. Additionally, strict, protocol‐driven management of hemoglobin and iron parameters may limit generalizability to routine clinical practice. Therefore, real‐world evidence evaluating heart failure outcomes associated with HIF‐PHIs in clinically vulnerable non‐dialysis CKD populations remains scarce. Patients with CKD frequently have concomitant heart failure and require diuretic therapy, making the cardiovascular safety of renal anemia treatment crucial in this high‐risk population. Nevertheless, comparative real‐world evidence regarding heart failure outcomes associated with HIF‐PHIs versus ESAs in non‐dialysis CKD patients with heart failure remains scarce. Therefore, we conducted a nationwide claims‐based cohort study using a large Japanese healthcare database to evaluate the risk of heart failure hospitalization associated with HIF‐PHI use compared with ESA use in this population.

## 2 Methods

### 2.1 Data Sources

We analyzed data obtained from the DeSC database (DeSC Healthcare Inc., Tokyo, Japan), which is a nationwide claims database in Japan. The database covers approximately 12 million insured individuals across several public health insurance schemes and has an age distribution similar to that of the general Japanese population [10, 11].

### 2.2 Study Design

We conducted a retrospective active‐comparator cohort study including patients with heart failure who newly initiated HIF‐PHIs or ESAs during ongoing diuretic therapy between August 1, 2020, and March 31, 2025. Heart failure was defined by at least one recorded diagnosis code (ICD‐10: I110 or I50, excluding “suspected” flags) prior to the index date. ESAs served as the active comparator given their shared indication for renal anemia and established use in head‐to‐head trials [4, 9]. Eligible patients were adults (aged ≥ 18 years) requiring ≥ 180 days of baseline continuous enrollment, with no history of dialysis or HIF‐PHIs/ESAs use during this period. The index date was the first prescription date. Primary analysis, we estimated inverse probability of treatment weighting (IPTW)‐adjusted HRs for heart failure hospitalization. Secondary outcomes were evaluated by estimating IPTW‐adjusted HRs for emergency heart failure hospitalization and hospitalization accompanied by intravenous diuretic administration. This study was designed to emulate a hypothetical target trial comparing initiation of HIF‐PHIs versus ESAs among patients with heart failure receiving diuretics. The specification of the hypothetical target trial and its emulation in the present study are summarized in Table S1, following the TARGET reporting recommendations [12].

### 2.3 Exposure

Target drugs (HIF‐PHIs and ESAs) were identified using anatomical therapeutic chemistry (ATC) classification codes (Table S2). For non‐roxadustat HIF‐PHIs, exposure duration was defined by the prescription days supply, excluding as‐needed use. For roxadustat, administered three times weekly, duration was estimated from the prescribed dosing frequency. Given variable ESA dosing intervals, each ESA administration was assumed to provide 30 days of treatment coverage. To allow for delays in prescription refills and follow‐up visits, treatment episodes were extended using a 30‐day grace period. Continuous treatment was defined when the subsequent prescription was dispensed within 30 days after the end of the previous prescription's estimated supply.

### 2.4 Definition of Covariates and Outcome

Propensity scores were derived using 19 baseline covariates that were selected to reflect both treatment selection between HIF‐PHIs and ESAs and established risk factors for heart failure outcomes [13, 14, 15, 16]. The covariates comprised demographic and healthcare utilization variables (age, sex, and calendar year), Charlson comorbidity index (CCI) calculated based on multiple comorbidities [17], key clinical comorbidities (such as hypertension, dyslipidemia, and atrial fibrillation), and selected concomitant medications (such as renin angiotensin inhibitors (RASIs), diuretics, and sodium–glucose cotransporter‐2 (SGLT2) inhibitors). Definitions of all covariates and the corresponding code lists are presented in Tables S3 and S4. Calendar year at the index date was entered into the propensity score model as an ordinal variable to account for temporal changes in patient characteristics and prescribing patterns. Baseline comorbidities were defined using diagnoses recorded from 6 months prior to the index month through the index month. Diagnoses recorded in the index month were included only if the recorded diagnosis date preceded the index date.

The primary outcome of this study was hospitalization in which the principal diagnosis or the condition leading to admission was heart failure. The secondary outcome was emergency hospitalization for heart failure as the primary diagnosis or the reason for admission and hospitalization with intravenous diuretic administration; emergency hospitalization was defined using the Japanese procedure codes A205 or A300. Additional endpoints included discontinuation of the target drug, initiation of the control drug, undergoing dialysis, and withdrawal from the database. Dialysis initiation was identified using the Japanese procedure codes J038 and C102, with the first recorded procedure date regarded as the dialysis initiation date.

### 2.5 Statistical Analysis

A multivariable logistic regression model was used to estimate propensity scores based on the 19 baseline covariates described above. Covariate balance between the HIF‐PHI and ESA groups was achieved using IPTW. IPTW was selected as the primary propensity score method because the primary objective of this study was to estimate the average treatment effect of initiating HIF‐PHIs versus ESAs in the overall eligible population, rather than the treatment effect within a selected subgroup. This estimand aligned with the study objective of evaluating the comparative effects of HIF‐PHIs versus ESAs in routine clinical practice. To reduce the influence of extreme weights, stabilized IPTW was applied, and weights below the 1st percentile or above the 99th percentile were truncated at the corresponding percentile values. After weighting, baseline covariate balance was evaluated using standardized mean differences (SMDs). An SMD of < 0.10 was regarded as indicating optimal balance, whereas values < 0.25 were considered acceptable [18]. The distributions of the estimated propensity scores in the HIF‐PHI and ESA groups are presented in Figure S1 to illustrate the overlap of propensity scores between treatment groups. Incidence rates were calculated as the number of events per 1000 person‐years (PY). In the analysis, we performed an IPTW‐adjusted Cox hazard analysis to calculate the HR for outcome occurrence (the ratio of HIF‐PHIs to ESAs). In the on‐treatment analysis, follow‐up continued until the first occurrence of any of the following: the study outcome, discontinuation of the index treatment, initiation of the comparator treatment, initiation of dialysis, or withdrawal from the database. Statistical analyses were conducted using R version 4.5.3 (R Foundation for Statistical Computing, Vienna, Austria).

### 2.6 Subgroup Analyses

Three prespecified subgroup analyses were conducted to examine potential effect modification. Within each subgroup, propensity scores were re‐estimated using the same covariates as those included in the primary analysis, and IPTW was subsequently applied to achieve covariate balance. Outcome risks were then evaluated using IPTW‐adjusted Cox proportional hazards models, and HRs with 95% CIs were estimated. The predefined subgroups were as follows:

1. Age ≥ 75 versus age < 75 years.
2. CCI ≥ 5 versus CCI < 5.
3. Type of HIF‐PHIs (daprodustat, enarodustat, molidustat, roxadustat, or vadadustat).

The age threshold of 75 years was chosen because it corresponds to the eligibility age for Japan's medical care system for older adults, where the health insurance scheme and healthcare delivery system differ from those for younger individuals.

### 2.7 Sensitivity Analyses

To examine the robustness of the primary findings, four sensitivity analyses were prespecified.

1. Propensity score matching (PSM): Patients were matched 1:1 using nearest‐neighbor matching on the logit of the propensity score with a caliper of 0.2 standard deviations of the logit. Covariate balance in the matched cohort was evaluated using SMD, and outcome HRs were estimated using Cox proportional hazards models.
2. Competing risk analysis: HRs accounting for death as a competing risk were estimated using Fine–Gray subdistribution hazard models adjusted for propensity scores, restricted to patients for whom mortality information was available.
3. Analysis with health checkup data: Among patients with available health checkup data within 1 year before the index date, an analysis identical to the primary analysis was performed using propensity scores additionally adjusted for health‐check parameters (body mass index [BMI], estimated glomerular filtration rate [eGFR], hemoglobin, and systolic blood pressure), demographic and healthcare utilization variables (age, sex, and calendar year), CCI calculated based on multiple comorbidities, comorbidities (dyslipidemia and atrial fibrillation), and selected concomitant medications (including RASIs, loop diuretics, beta blockers, and SGLT2 inhibitors).
4. Intention‐to‐treat (ITT) analysis: An identical analysis to the primary analysis was repeated using an ITT approach, in which follow‐up continued until outcome occurrence or withdrawal from the database regardless of treatment discontinuation or switching.

### 2.8 Ethics

This study adhered to the principles of the Declaration of Helsinki. Under the Japanese Ethical Guidelines for Medical and Biological Research Involving Human Subjects, studies using fully anonymized secondary data are exempt from institutional review board approval and the requirement for informed consent. Accordingly, neither institutional review board approval nor informed consent was required for the present study.

## 3 Results

**FIGURE 1:**
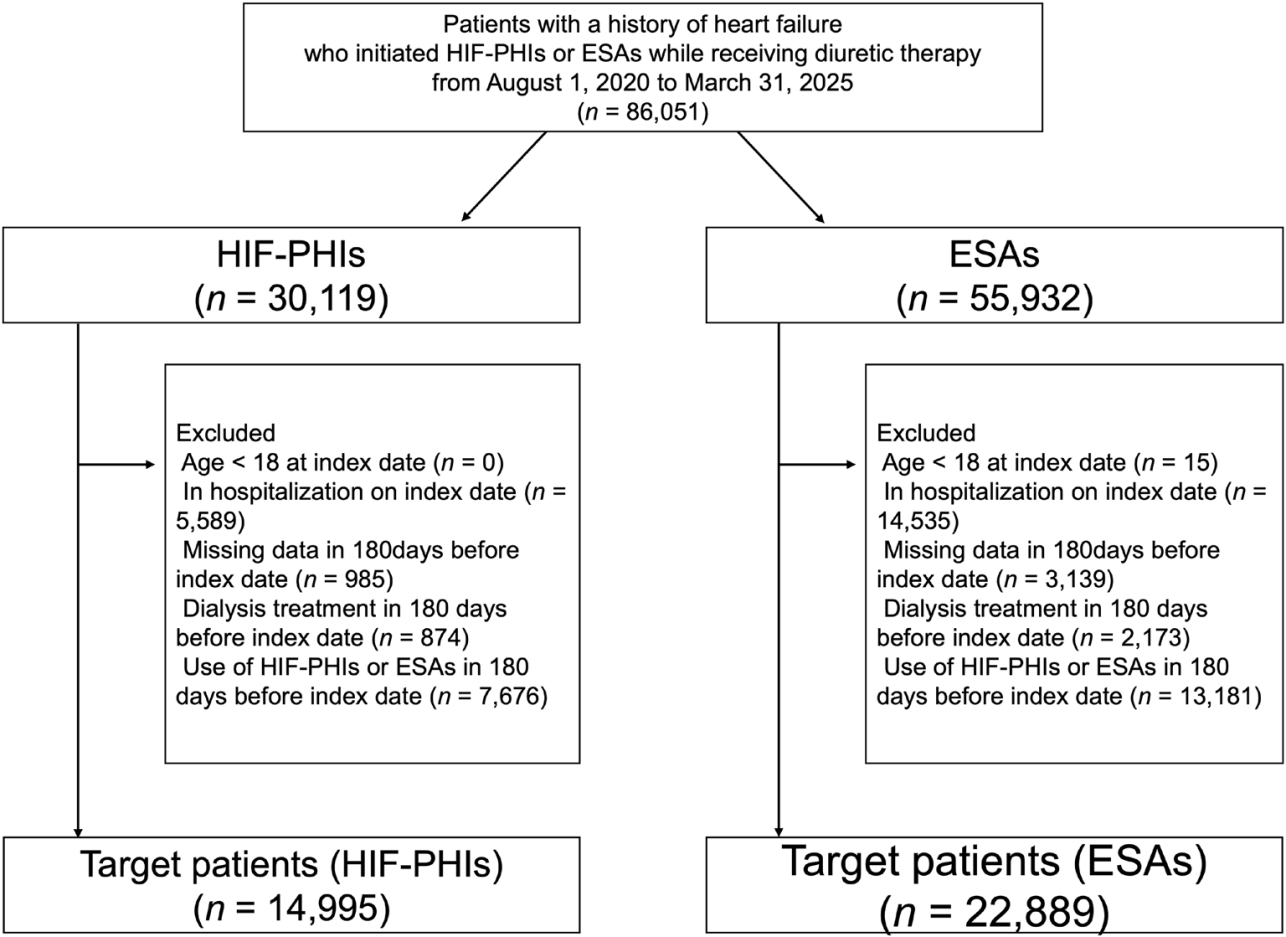
Flowchart of target patient inclusion. The index date was defined as the date of the first HIF‐PHIs or ESAs dispensation. ESAs, erythropoiesis‐stimulating agents; HIF‐PHIs, hypoxia‐inducible factor prolyl hydroxylase inhibitors.

**FIGURE 2:**
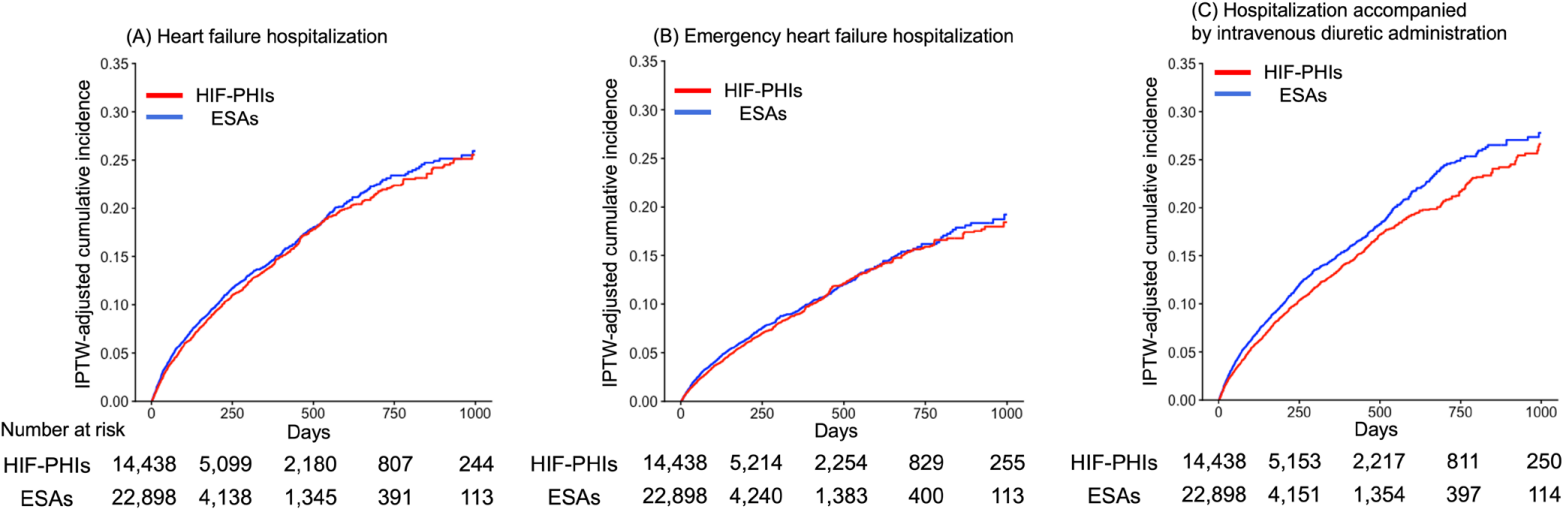
The IPTW‐adjusted cumulative incidence curves for outcomes. IPTW‐adjusted cumulative incidence curves for (A) heart failure hospitalization, (B) emergency heart failure hospitalization, and (C) hospitalization with intravenous diuretics. ESAs, erythropoiesis‐stimulating agents; HIF‐PHIs, hypoxia‐inducible factor prolyl hydroxylase inhibitors; IPTW, inverse probability of treatment weighting.

The study cohort consisted of 14 995 HIF‐PHI users and 22 889 ESA users (Figure 1). Baseline characteristics before and after IPTW are presented in Table S5. Before weighting, imbalances were observed in several baseline covariates, including age, calendar year, CCI, and concomitant medication use. After stabilization and truncation of IPTW at the 1st and 99th percentiles, covariate balance improved substantially, with most post‐IPTW SMDs falling below 0.10 and all remaining below 0.25. The estimated propensity score distributions demonstrated substantial overlap between the treatment groups (Figure S1). The results for the primary and secondary outcomes are presented in Table 1. For heart failure hospitalization, the incidence rate following IPTW adjustment was 157.1 per 1000 PY in the HIF‐PHIs cohort and 200.4 per 1000 PY in the ESAs cohort. The IPTW‐adjusted HR was 0.93 (95% CI [0.87–1.01]). For emergency heart failure hospitalization and hospitalization with intravenous diuretic administration, HRs were 0.94 (95% CI [0.84–1.03]) and 0.87 (95% CI [0.81–0.94]). The IPTW‐adjusted cumulative incidence curves for outcomes are illustrated in Figure 2.

**TABLE 1:** Incidence rates and HRs for primary and secondary outcomes.

Outcomes	Crude			IPTW‐adjusted		
	The incidence rate in HIF‐PHIs patients (*n* = 14 995) (/1000 PY)	The incidence rate in ESAs patients (*n* = 22 889) (/1000 PY)	HR [95% CI]	The incidence rate in HIF–PHIs patients (weighted *n* = 14 438) (/1000 PY)	The incidence rate in ESAs patients (weighted *n* = 22 898) (/1000 PY)	HR [95% CI]
Heart failure hospitalization	164.9	178.7	1.01 [0.94–1.08]	157.8	177.0	0.93 [0.87–1.01]
Emergency heart failure hospitalization	104.9	113.3	1.00 [0.92–1.09]	101.7	111.6	0.94 [0.85–1.03]
Hospitalization accompanied by intravenous diuretic administration	152.6	186.4	0.89 [0.83–0.95]	151.7	179.4	0.87 [0.81–0.94]

Abbreviations: CI, confidence interval; ESAs, erythropoiesis‐stimulating agents; HIF‐PHIs, hypoxia‐inducible factor prolyl hydroxylase inhibitors; HR, hazard ratio; IPTW, inverse probability of treatment weighting; PY, person‐years.

### 3.1 Subgroup Analysis

Results of the subgroup analyses are presented in Table 2. The association between HIF‐PHIs and heart failure hospitalization was generally consistent with that observed in the primary analysis. No substantial increase in risk associated with HIF‐PHIs use was observed across subgroups defined by age, CCI, or HIF‐PHI type. HRs were generally close to unity, and all 95% CIs included 1.0.

**TABLE 2:** Subgroup analyses for primary and secondary outcomes.

Subgroups	HIF‐PHIs patients (*n*)	ESAs patients (*n*)	IPTW‐adjusted HR [95% CI]		
			Heart failure hospitalizations	Emergency heart failure hospitalization	Hospitalization with intravenous diuretic administration
Overall	14 995	22 889	0.93 [0.87–1.01]	0.94 [0.85–1.03]	0.87 [0.81–0.94]
Age					
< 75 years old	885	2115	0.92 [0.65–1.29]	0.78 [0.52–1.17]	0.75 [0.56–1.02]
≥ 75 years old	14 110	20 774	0.95 [0.88–1.02]	0.96 [0.87–1.06]	0.89 [0.83–0.97]
CCI (excluding age)					
< 5	5479	6823	0.95 [0.83–1.08]	0.93 [0.79–1.10]	0.95 [0.83–1.08]
≥ 5	9516	16 066	0.94 [0.86–1.03]	0.96 [0.86–1.07]	0.85 [0.78–0.93]
HIF‐PHI type					
Daprodustat	9927	22 889	0.94 [0.86–1.03]	0.95 [0.86–1.06]	0.88 [0.81–0.97]
Enarodustat	251	22 889	0.71 [0.42–1.20]	0.78 [0.41–1.50]	0.80 [0.49–1.30]
Molidustat	783	22 889	1.17 [0.92–1.50]	1.36 [1.02–1.82]	1.17 [0.91–1.50]
Roxadustat	1423	22 889	0.97 [0.81–1.15]	0.98 [0.79–1.22]	0.90 [0.75–1.08]
Vadadustat	2607	22 889	0.91 [0.79–1.06]	0.85 [0.71–1.03]	0.78 [0.67–0.91]

*Note:* For analyses according to HIF‐PHI type, each HIF‐PHI subgroup was compared with the overall ESA cohort.

Abbreviations: CCI, Charlson comorbidity index; ESAs, erythropoiesis‐stimulating agents; HIF‐PHIs, hypoxia‐inducible factor prolyl hydroxylase inhibitors; HR, hazard ratio; IPTW, inverse probability of treatment weighting.

### 3.2 Sensitivity Analysis

#### 3.2.1 PSM

Analysis of the propensity score‐matched cohort (14 005 matched pairs; baseline characteristics are presented in Table S6) yielded findings similar to those of the primary analysis. The HR for heart failure hospitalization was 0.96 (95% CI 0.89–1.04) (Table S7).

#### 3.2.2 Competing Risk Analysis

Among patients with available mortality information, Fine–Gray subdistribution hazard models accounting for death as a competing risk were generally consistent with those of the primary analysis. The HR for heart failure hospitalization was 0.86 (95% CI 0.78–0.95) (Table S7). To further examine whether the competing‐risk results could have been influenced by differential mortality between treatment groups, we additionally evaluated all‐cause mortality during the on‐treatment follow‐up. The incidence of all‐cause mortality was lower among HIF‐PHI users than among ESA users (Table S8), with an IPTW‐adjusted HR of 0.38 (95% CI 0.34–0.43).

#### 3.2.3 Analysis With Health Checkup Data

Among patients with available health checkup data, the results remained generally consistent with those of the primary analysis after additional adjustment for clinical parameters, including BMI, eGFR, hemoglobin, and systolic blood pressure (Table S9). The HR for heart failure hospitalization was 0.87 [95% CI 0.58–1.29] (Table S7).

#### 3.2.4 ITT Analysis

In the ITT analysis, the association between HIF‐PHIs and heart failure hospitalization remained generally consistent with that observed in the primary analysis, although the estimated HR reached statistical significance (HR for heart failure hospitalization: 0.93; 95% CI [0.88–0.99]) (Table S7).

## 4 Discussion

In this nationwide, claims‐based cohort study of non‐dialysis patients with heart failure receiving diuretic therapy, HIF‐PHIs use was not associated with a clear increase in the risk of heart failure hospitalization compared with ESA use. Similar findings were observed for emergency heart failure hospitalization and hospitalization accompanied by intravenous diuretic administration. Furthermore, the results were generally consistent across subgroup and multiple sensitivity analyses, supporting the robustness of our findings.

Our findings complement and extend evidence from the ASCEND clinical trial program. In a recent secondary analysis of the ASCEND‐ND and ASCEND‐D trials [7], daprodustat was associated with a numerically higher risk of first heart failure hospitalization among non‐dialysis patients (HR 1.22; 95% CI 0.95–1.56). Such association appeared stronger among patients with a history of heart failure (HR 1.37; 95% CI 0.89–2.11). Furthermore, daprodustat was associated with an increased risk of total (first and recurrent) heart failure hospitalizations in non‐dialysis patients (HR 1.46; 95% CI 1.11–1.92). In contrast, the estimated HR for heart failure hospitalization in the present study was 0.93 (95% CI 0.87–1.01). Several factors may explain these differences. First, heart failure outcomes in the ASCEND trials were evaluated as secondary analyses rather than prespecified primary endpoints, and the number of heart failure events was relatively limited [7]. Second, important differences existed in the study populations. While the ASCEND trials enrolled patients with CKD and anemia under protocol‐driven conditions, individuals with severe heart failure, recent cardiovascular events, or other conditions that could interfere with trial participation were generally excluded [4, 9]. In contrast, our study specifically focused on patients with pre‐existing heart failure who were actively receiving diuretic therapy, thereby capturing a clinically vulnerable population frequently encountered in routine clinical practice. Therefore, our findings provide complementary real‐world evidence regarding the cardiovascular safety of HIF‐PHIs in patients at particularly high risk of worsening heart failure. Finally, treatment and monitoring strategies may differ substantially between randomized trials and routine care. In the ASCEND program, hemoglobin targets, dose adjustments, and anemia management were protocolized [4, 9], whereas treatment selection and monitoring in clinical practice are individualized according to patient characteristics and physician judgment. Such differences may influence observed cardiovascular outcomes and should be considered when interpreting the findings of both studies. In contrast to the concerns raised by the ASCEND analyses, several smaller observational studies from Japan have reported favorable findings regarding HIF‐PHI use in patients with heart failure and renal anemia. Some studies found no apparent worsening of heart failure during short‐term HIF‐PHI treatment [19, 20], whereas others reported improvements in heart failure‐related clinical parameters following initiation of HIF‐PHI [21, 22]. Despite important methodological limitations, including small sample sizes, short observation periods, and the absence of comparator groups, these studies yielded findings broadly consistent with the present results.

Among the secondary outcomes, hospitalization accompanied by intravenous diuretic administration was significantly less frequent in the HIF‐PHI cohort than in the ESA cohort. However, this finding was not consistently observed across other heart failure‐related endpoints, such as the primary outcome of heart failure hospitalization and emergency heart failure hospitalization. Similarly, while an increased risk of emergency heart failure hospitalization was observed in the molidustat subgroup, the point estimates for the primary outcome and hospitalization accompanied by intravenous diuretic administration were also directionally consistent, although they did not reach statistical significance. Given the relatively small number of patients in the molidustat subgroup and the exploratory nature of the subgroup analyses, these findings should be interpreted with caution and may represent chance findings due to multiple comparisons.

Potential mechanisms linking HIF‐PHIs to heart failure have been proposed, including the activation of vascular endothelial growth factor pathways, alterations in sodium and fluid handling, and interactions with the renin–angiotensin–aldosterone system [6, 7, 8]. These concerns have contributed to ongoing discussion regarding the cardiovascular safety of HIF‐PHIs, particularly among patients with established heart failure. However, in the present study, which included a substantial number of patients with pre‐existing heart failure receiving diuretic therapy, we observed no evidence of a clinically meaningful increase in heart failure‐related hospitalization. Although the present study cannot directly address biological mechanisms, our findings suggest that any potential adverse cardiovascular effects of HIF‐PHIs may not translate into a substantial increase in the risk of heart failure hospitalization in routine clinical practice.

Several limitations should be acknowledged. First, residual confounding from unmeasured clinical factors cannot be excluded. Important clinical information related to heart failure severity, including left ventricular ejection fraction, New York Heart Association functional class, detailed laboratory data, and physician prescribing preferences, was unavailable in the claims database. Although laboratory measures of iron status, such as transferrin saturation and ferritin, were unavailable, baseline iron supplementation was included in the propensity score model as a proxy for the clinical management of iron deficiency. Nevertheless, residual confounding related to the severity of iron deficiency cannot be completely excluded. Furthermore, although sensitivity analyses incorporating available health checkup data yielded similar findings, detailed laboratory information was not available for all patients, and residual confounding due to unavailable clinical variables cannot be entirely ruled out. Furthermore, although calendar year was included in the propensity score model, evolving prescribing practices after the introduction of HIF‐PHIs may not have been fully captured, and residual calendar‐time‐related bias cannot be completely excluded. The substantially lower all‐cause mortality observed among HIF‐PHI users also suggests that residual confounding or differences in patient characteristics between treatment groups may have remained despite propensity score adjustment, as previous studies have generally not reported such a large mortality difference between HIF‐PHIs and ESAs [23]. Second, outcomes and comorbidities were identified using administrative claims data and may have been subject to misclassification [24]. However, our primary outcome was defined using hospitalization records accompanied by a principal diagnosis or condition leading to admission for heart failure, which may have improved outcome specificity compared with using diagnosis codes alone. In addition, because Japan operates a universal health insurance system, hospitalizations and medical procedures are comprehensively captured through reimbursement claims, reducing the likelihood of substantial underascertainment of hospitalized events. Third, our primary analysis adopted an on‐treatment approach using baseline propensity score weighting. Because treatment discontinuation or switching may be related to changes in clinical status, informative censoring cannot be completely excluded. In addition, treatment decisions during follow‐up may have been influenced by evolving clinical factors, such as hemoglobin levels, kidney function, and overall clinical status, which may also affect subsequent outcomes. Therefore, baseline propensity score weighting cannot fully account for time‐varying confounding. Although methods such as inverse probability of censoring weighting and marginal structural models may reduce these potential biases, such analyses were beyond the scope of the present study. Nevertheless, the intention‐to‐treat sensitivity analysis yielded generally consistent results, providing some reassurance regarding the robustness of the findings. Fourth, differences in treatment administration between HIF‐PHIs and ESAs may have influenced actual treatment exposure. HIF‐PHIs are orally administered, whereas ESAs are administered by injection, which may result in differences in medication adherence and treatment persistence. Because claims data capture prescriptions and administrations rather than actual medication consumption, differential exposure misclassification between treatment groups cannot be excluded. Given that approximately one‐fifth of the study population had dementia, differences in adherence between treatment modalities may be particularly relevant. Finally, because this study used Japanese claims data, the generalizability of our findings to other healthcare systems and populations should be evaluated in future studies.

In this nationwide, real‐world cohort study of non‐dialysis patients with heart failure receiving diuretic therapy, HIF‐PHI use was not associated with a clear increase in the risk of heart failure hospitalization compared with ESA use. The findings were generally consistent across secondary outcomes, subgroup analyses, and sensitivity analyses. These results provide complementary real‐world evidence regarding heart failure‐related outcomes associated with HIF‐PHI use among patients with heart failure receiving diuretic therapy.

## Author Contributions

Conception and design: Yuki Kunitsu. Data analysis/interpretation: Yuki Kunitsu, Keiko Ikuta, Daiki Hira, Shunsaku Nakagawa, and Tomohiro Terada. Statistical analysis: Yuki Kunitsu. Manuscript drafting: Yuki Kunitsu. Critical revision of the manuscript: Keiko Ikuta, Daiki Hira, Shunsaku Nakagawa, and Tomohiro Terada. Each author contributed important intellectual content during manuscript drafting or revision, agreed to be personally accountable for their own contributions, and ensured that questions pertaining to the accuracy or integrity of any portion of the work, even those in which the author was not directly involved, were appropriately investigated and resolved, with documentation in the literature, if appropriate.

## Funding

This study was supported by JSPS KAKENHI (grant number: JP25K18676). The funding source played no role in the study design, data collection, analysis, reporting, or decision to submit the manuscript for publication.

## Ethics Statement

This retrospective cohort study was conducted using anonymized data from the DeSC claims database (Tokyo, Japan). According to the Japanese Ethical Guidelines for Medical and Biological Research Involving Human Subjects, the use of fully anonymized secondary data is exempt from institutional review board approval and informed consent. Consequently, ethics approval and consent to participate were not required for this study.

## Consent

The authors have nothing to report.

## Conflicts of Interest

T.T. has received speaker honoraria from Chugai Pharmaceutical Co. Ltd. The other authors declare no conflicts of interest.

## Supporting information


**Table S1:** Definition of HIF‐PHIs and ESAs.
**Table S2:** Definition of diseases based on the International Classification of Diseases, 10th edition (ICD‐10) diagnosis codes.
**Table S3:** Definitions of drugs based on ATC codes.
**Table S4:** Baseline characteristics of HIF‐PHIs and ESAs cohorts before and after IPTW.
**Table S5:** Results of sensitivity analyses for primary and secondary outcomes.
**Table S6:** Baseline characteristics after propensity score matching.
**Table S7:** All‐cause mortality during on‐treatment follow‐up.
**Table S8:** Baseline characteristics of HIF‐PHIs and ESAs health checkup cohorts before and after IPTW.
**Figure S1:** Propensity score distributions in the HIF‐PHIs and ESAs groups.

## Data Availability

The data that support the findings of this study are available from DeSC Healthcare Inc.; however, restrictions apply to the availability of these data, which were used under license for the current study and are therefore not publicly available. Data access may be requested directly from DeSC Healthcare Inc., subject to their approval and data use agreements.
