# Supplementary material for: Risk of Heart Failure Hospitalization Associated With Hypoxia‐Inducible Factor Prolyl Hydroxylase Inhibitors Versus Erythropoiesis‐Stimulating Agents: A Nationwide Claims‐Based Cohort Study
Source: Pharmacoepidemiol Drug Saf. 2026 Aug 29;35(9):e70453. doi: 10.1002/pds.70453 (PMC13525448; doi:10.1002/pds.70453)
Supplement: Supplementary file 1 [file PDS-35-e70453-s001.pdf]

Supplementary Materials

Risk of heart failure hospitalization associated with hypoxia-inducible factor prolyl hydroxylase inhibitors versus erythropoiesis-stimulating agents: A nationwide claims-based cohort study

Pharmacoepidemiology and Drug Safety

Authors: Yuki Kunitsu, Keiko Ikuta, Daiki Hira, Shunsaku Nakagawa, Tomohiro Terada

Corresponding author: Yuki Kunitsu
Department of Clinical Pharmacology and Therapeutics, Kyoto University Hospital, 54 Shogoin-Kawahara-cho, Sakyo-ku, Kyoto, 606-8507, Japan
Email: ykunitsu@kuhp.kyoto-u.ac.jp

Supplementary Table S1. Definition of HIF-PHIs and ESAs
Supplementary Table S2. Definition of diseases based on the International Classification of Diseases, 10th edition (ICD-10) diagnosis codes
Supplementary Table S3. Definitions of drugs based on ATC codes
Supplementary Table S4. Baseline characteristics of HIF-PHIs and ESAs cohorts before and after IPTW
Supplementary Table S5. Results of sensitivity analyses for primary and secondary outcomes
Supplementary Table S6. Baseline characteristics after propensity score matching
Supplementary Table S7. All-cause mortality during on-treatment follow-up
Supplementary Table S8. Baseline characteristics of HIF-PHIs and ESAs health checkup cohorts before and after IPTW
Supplementary Figure S1. Propensity score distributions in the HIF-PHIs and ESAs groups

Supplementary Table S1. Definition of HIF-PHIs and ESAs

Category	Based ATC codes	Drug name
HIF-PHIs	B03XA	Daprodustat, Enarodustat, Molidustat sodium, Roxadustat, and Vadadustat
ESAs	B03XA	epoetin alfa, epoetin beta, epoetin beta pegol, epoetin kappa, and darbepoetin alfa

ATC, anatomical therapeutic chemistry. ESAs, erythropoiesis-stimulating agents. HIF-PHIs, hypoxia-inducible factor prolyl hydroxylase inhibitors.

Supplementary Table S2. Definition of diseases based on the International Classification of Diseases, 10th Edition (ICD-10) diagnosis codes

Diseases	ICD-10 codes
Anemia	D50−64
Renal anemia	N19 and D63.8 (both required)
Atrial fibrillation	I48
Dyslipidemia	E78
Hypertension	I10−15
Hypotension	I95
For CCI	
AIDS/HIV	B20–B22, B24
Any malignancy	C00–C26, C30–C34, C37–C41, C43, C45–C58, C60–C76, C81–C85, C88, C90–C97
Cerebrovascular disease	G45, G46, H34.0, I60–I69
Chronic pulmonary disease	I27.8, I27.9, J40–J47, J60–J67, J68.4, J70.1, J70.3
Congestive heart failure	I09.9, I11.0, I13.0, I13.2, I25.5, I42.0, I42.5–I42.9, I43, I50, P29.0
Dementia	F00–F03, F05.1, G30, G31.1
Diabetes with chronic complication	E10.2–E10.5, E10.7, E11.2–E11.5, E11.7, E12.2–E12.5, E12.7, E13.2– E13.5, E13.7, E14.2–E14.5, E14.7
Diabetes without chronic complication	E10.0, E10.1, E10.6, E10.8, E10.9, E11.0, E11.1, E11.6, E11.8, E11.9, E12.0, E12.1, E12.6, E12.8, E12.9, E13.0, E13.1, E13.6, E13.8, E13.9, E14.0, E14.1, E14.6, E14.8, E14.9
Hemiplegia or paraplegia	G04.1, G11.4, G80.1, G80.2, G81, G82, G83.0–G83.4, G83.9
Metastatic solid tumor	C77–C80
Mild liver disease	B18, K70.0–K70.3, K70.9, K71.3–K71.5, K71.7, K73, K74, K76.0, K76.2–K76.4, K76.8, K76.9, Z94.4
Moderate or severe liver disease	I85.0, I85.9, I86.4, I98.2, K70.4, K71.1, K72.1, K72.9, K76.5, K76.6, K76.7
Myocardial infarction	I21, I22, I25.2
Peptic ulcer disease	K25–K28
Peripheral vascular disease	I70, I71, I73.1, I73.8, I73.9, I77.1, I79.0, I79.2, K55.1, K55.8, K55.9, Z95.8, Z95.9

Diseases	ICD-10 codes
Renal disease	I12.0, I13.1, N03.2–N03.7, N05.2–N05.7, N18, N19, N25.0, Z49.0–Z49.2, Z94.0, Z99.2
Rheumatic disease	M05, M06, M31.5, M32–M34, M35.1, M35.3, M36.0

CCI, Charlson comorbidity index

Supplementary Table S3. Definitions of drugs based on ATC codes

Class name	ATC codes
Beta blockers	C07
Calcium channel blockers	C08C
Diuretics	C03
Loop diuretics	C03CA
Potassium-sparing diuretics	C03D
Thiazide and thiazide-like diuretics	C03AA, C03BA
Vasopressin V2 receptor antagonists	C03XA
Iron preparations	B03
Non-topical NSAIDs	M01A
RASIs	C09
Angiotensin receptor neprilysin inhibitors	C09D9
SGLT2 inhibitors	A10BK, A10BD

The compounded drugs containing these ingredients were also used in the analysis.

ATC, anatomical therapeutic chemistry. RASIs, renin-angiotensin system inhibitors. SGLT2, sodium–glucose cotransporter-2. NSAIDs, non-steroidal anti-inflammatory drugs.

Supplementary Table S4. Baseline characteristics of HIF-PHIs and ESAs cohorts before and after IPTW

Characteristics	Crude			IPTW-adjusted		
	HIF-PHIs patients (n = 14,995)	ESAs patients (n = 22,889)	SMD	HIF-PHIs patients (weighted n = 14,438)	ESAs patients (weighted n = 22,898)	SMD
Male[a], n (%)	6,321 (42.2)	10,810 (47.2)	0.10	6,402 (44.3)	10,325 (45.1)	0.02
Age[a], years, average (SD)	86.0 (7.25)	84.7 (8.20)	0.17	85.4 (7.60)	85.2 (7.97)	0.02
Calendar year on index date[a], n (%)						
2020	352 (2.3)	3,671 (16.0)	0.49	1,041 (7.2)	2,430 (10.6)	0.12
2021	2,759 (18.4)	8,155 (35.6)	0.40	4,302 (29.8)	6,587 (28.8)	0.02
2022	5,220 (34.8)	5,862 (25.6)	0.20	4,384 (30.4)	6,705 (29.3)	0.02
2023	4,460 (29.7)	3,825 (16.7)	0.31	3,286 (22.8)	5,018 (21.9)	0.02
2024	1,901 (12.7)	1,137 (5.0)	0.27	1,209 (8.4)	1,828 (8.0)	0.01
2025	303 (2.0)	239 (1.0)	0.08	215 (1.5)	329 (1.4)	<0.01
Comorbidities, n (%)						
Anemia	14,712 (98.1)	22,665 (99.0)	0.08	14,182 (98.2)	22,663 (99.0)	0.06
Renal anemia	14,206 (94.7)	21,967 (96.0)	0.06	13,706 (94.9)	21,914 (95.7)	0.04
Atrial fibrillation[a]	6,086 (40.6)	8,226 (35.9)	0.10	5,391 (37.3)	8,597 (37.5)	<0.01
Dyslipidemia[a]	9,080 (60.6)	13,957 (61.0)	0.01	8,742 (60.5)	13,934 (60.9)	0.01
Hypertension[a]	14,145 (94.3)	21,610 (94.4)	<0.01	13,606 (94.2)	21,601 (94.3)	<0.01
Hypotension[a]	206 (1.4)	366 (1.6)	0.02	207 (1.4)	343 (1.5)	0.01
For CCI						
AIDS/HIV	1 (0.0)	16 (0.1)	0.03	2 (0.0)	12 (0.1)	0.02

Characteristics	Crude					IPTW-adjusted				
	HIF-PHIs patients (n = 14,995)		ESAs patients (n = 22,889)		SMD	HIF-PHIs patients (weighted n = 14,438)		ESAs patients (weighted n = 22,898)		SMD
Any malignancy	2,691	(17.9)	5,099	(22.3)	0.11	2,804	(19.4)	4,883	(21.3)	0.05
Cerebrovascular disease	5,266	(35.1)	8,742	(38.2)	0.06	5,365	(37.2)	8,487	(37.1)	<0.01
Chronic pulmonary disease	4,472	(29.8)	7,404	(32.3)	0.06	4,524	(31.3)	7,246	(31.6)	0.01
Congestive heart failure	14,414	(96.1)	22,001	(96.1)	<0.01	13,862	(96.0)	22,016	(96.1)	0.01
Dementia	3,437	(22.9)	5,199	(22.7)	0.01	3,388	(23.5)	5,161	(22.5)	0.02
Diabetes with chronic complication	2,293	(15.3)	4,557	(19.9)	0.12	2,439	(16.9)	4,187	(18.3)	0.04
Diabetes without chronic complication	1,568	(10.5)	2,738	(12.0)	0.05	1,529	(10.6)	2,629	(11.5)	0.03
Hemiplegia or paraplegia	203	(1.4)	445	(1.9)	0.05	234	(1.6)	402	(1.8)	0.01
Metastatic solid tumor	273	(1.8)	600	(2.6)	0.05	376	(2.6)	496	(2.2)	0.03
Mild liver disease	3,281	(21.9)	5,757	(25.2)	0.08	3,404	(23.6)	5,557	(24.3)	0.02
Moderate or severe liver disease	137	(0.9)	281	(1.2)	0.03	158	(1.1)	241	(1.1)	<0.01
Myocardial infarction	1,666	(11.1)	2,468	(10.8)	0.01	1,636	(11.3)	2,377	(10.4)	0.03
Peptic ulcer disease	3,640	(24.3)	6,325	(27.6)	0.08	3,684	(25.5)	6,039	(26.4)	0.02
Peripheral vascular disease	3,401	(22.7)	5,805	(25.4)	0.06	3,452	(23.9)	5,577	(24.4)	0.01
Renal disease	14,573	(97.2)	22,339	(97.6)	0.03	14,058	(97.4)	22,278	(97.3)	0.01
Rheumatic disease	963	(6.4)	1,519	(6.6)	0.01	964	(6.7)	1,464	(6.4)	0.01
CCI (excluding age) [a], average (SD)	5.34	(1.91)	5.69	(2.03)	0.18	5.52	(2.02)	5.56	(1.98)	0.02
Concomitant medication, n (%)										
Beta blockers[a]	6,593	(44.0)	9,259	(40.5)	0.07	5,975	(41.4)	9,558	(41.7)	0.01

Characteristics	Crude					IPTW-adjusted				
	HIF-PHIs patients (n = 14,995)		ESAs patients (n = 22,889)		SMD	HIF-PHIs patients (weighted n = 14,438)		ESAs patients (weighted n = 22,898)		SMD
Calcium channel blockers[a]	7,547	(50.3)	12,529	(54.7)	0.09	7,643	(52.9)	12,146	(53.0)	<0.01
Diuretics										
Loop diuretics[a]	12,679	(84.6)	20,063	(87.7)	0.09	12,421	(86.0)	19,780	(86.4)	0.01
Potassium-sparing diuretics[a]	5,930	(39.5)	7,354	(32.1)	0.16	5,117	(35.4)	8,031	(35.1)	0.01
Thiazide and thiazide-like diuretics[a]	2,002	(13.4)	3,165	(13.8)	0.01	1,946	(13.5)	3,109	(13.6)	<0.01
Vasopressin V2 receptor antagonists[a]	2,907	(19.4)	4,056	(17.7)	0.04	2,596	(18.0)	4,184	(18.3)	0.01
Iron preparations[a]	5,090	(33.9)	6,439	(28.1)	0.13	4,382	(30.4)	6,932	(30.3)	<0.01
Non-topical NSAIDs[a]	1,505	(10.0)	2,226	(9.7)	0.01	1,429	(9.9)	2,256	(9.9)	<0.01
RASIs										
Angiotensin receptor neprilysin inhibitors[a]	2,478	(16.5)	1,407	(6.1)	0.33	1,569	(10.9)	2,379	(10.4)	0.02
Other RASIs[a]	7,533	(50.2)	12,162	(53.1)	0.06	7,563	(52.4)	11,923	(52.1)	0.01
SGLT2 inhibitors[a]	2,614	(17.4)	2,297	(10.0)	0.22	1,916	(13.3)	2,980	(13.0)	0.01

[a]Parameters used to calculate propensity scores.

CCI, Charlson comorbidity index. ESAs, erythropoiesis-stimulating agents. HIF-PHIs, hypoxia-inducible factor prolyl hydroxylase inhibitors. IPTW, inverse probability of treatment weighting. NSAIDs, non-steroidal anti-inflammatory drugs. RASIs, renin-angiotensin system inhibitors. SD, standard deviation. SGLT2, sodium–glucose cotransporter-2. SMD, standardized mean difference

Supplementary Table S5. Results of sensitivity analyses for primary and secondary outcomes

Analysis	IPTW-adjusted HR [95% CI]		
	Heart failure hospitalizations	Emergency heart failure hospitalization	Hospitalization with intravenous diuretic administration
Primary analysis	0.93 [0.87−1.01]	0.94 [0.85−1.03]	0.87 [0.81−0.94]
PSM	0.96 [0.89−1.04]	0.98 [0.88−1.08]	0.91 [0.84−0.99]
Competing risk analysis	0.86 [0.78−0.95]	1.02 [0.93−1.12]	0.96 [0.89−1.03]
Analysis with health checkup data	0.87 [0.58−1.29]	0.64 [0.38−1.08]	0.82 [0.56−1.18]
ITT analysis	0.93 [0.88−0.99]	0.95 [0.88−1.02]	0.95 [0.90−1.01]

Competing risk analysis was restricted to patients with available mortality information. Analysis with health checkup data was restricted to patients with available health checkup data.

CI, confidence interval. HR, hazard ratio. IPTW, inverse probability of treatment weighting. ITT, Intention to treat. PSM, propensity score matching.

Supplementary Table S6. Baseline characteristics after propensity score matching

Characteristics	HIF-PHIs patients (n = 14,005)		ESAs patients (n = 14,005)		SMD
Male[a], n (%)	5,988	(42.8)	6,247	(44.6)	0.04
Age[a], years, average (SD)	85.9	(7.29)	85.5	(7.67)	0.05
Calendar year on index date[a], n (%)					
2020	352	(2.5)	355	(2.5)	<0.01
2021	2,759	(19.7)	2,978	(21.3)	0.04
2022	5,022	(35.9)	5,535	(39.5)	0.08
2023	4,077	(29.1)	3,770	(26.9)	0.05
2024	1,522	(10.9)	1,137	(8.1)	0.09
2025	273	(1.9)	230	(1.6)	0.02
Comorbidities, n (%)					
Anemia	13,754	(98.2)	13,856	(98.9)	0.06
Renal anemia	13,301	(95.0)	13,407	(95.7)	0.04
Atrial fibrillation[a]	5,575	(39.8)	5,266	(37.6)	0.05
Dyslipidemia[a]	8,476	(60.5)	8,481	(60.6)	<0.01
Hypertension[a]	13,201	(94.3)	13,202	(94.3)	<0.01
Hypotension[a]	194	(1.4)	197	(1.4)	<0.01
For CCI					
AIDS/HIV	1	(0.0)	6	(0.0)	0.02
Any malignancy	2,565	(18.3)	2,979	(21.3)	0.07
Cerebrovascular disease	4,987	(35.6)	5,172	(36.9)	0.03
Chronic pulmonary disease	4,214	(30.1)	4,443	(31.7)	0.04
Congestive heart failure	13,450	(96.0)	13,460	(96.1)	<0.01
Dementia	3,242	(23.1)	3,232	(23.1)	<0.01
Diabetes with chronic complication	2,194	(15.7)	2,448	(17.5)	0.05
Diabetes without chronic complication	1,465	(10.5)	1,584	(11.3)	0.03
Hemiplegia or paraplegia	196	(1.4)	231	(1.6)	0.02
Metastatic solid tumor	268	(1.9)	247	(1.8)	0.01
Mild liver disease	3,095	(22.1)	3,384	(24.2)	0.05
Moderate or severe liver disease	131	(0.9)	129	(0.9)	<0.01
Myocardial infarction	1,566	(11.2)	1,421	(10.1)	0.03
Peptic ulcer disease	3,456	(24.7)	3,659	(26.1)	0.03
Peripheral vascular disease	3,215	(23.0)	3,391	(24.2)	0.03

Renal disease	13,637	(97.4)	13,625	(97.3)	0.01
Rheumatic disease	917	(6.5)	881	(6.3)	0.01
CCI (excluding age) [a], average (SD)	5.38	(1.92)	5.51	(1.92)	0.07
Concomitant medication, n (%)					
Beta blockers[a]	6,034	(43.1)	5,780	(41.3)	0.04
Calcium channel blockers[a]	7,167	(51.2)	7,446	(53.2)	0.04
Diuretics					
Loop diuretics[a]	11,917	(85.1)	12,048	(86.0)	0.03
Potassium-sparing diuretics[a]	5,334	(38.1)	4,958	(35.4)	0.06
Thiazide and thiazide-like diuretics[a]	1,894	(13.5)	1,861	(13.3)	0.01
Vasopressin V2 receptor antagonists[a]	2,624	(18.7)	2,504	(17.9)	0.02
Iron preparations[a]	4,637	(33.1)	4,294	(30.7)	0.05
Non-topical NSAIDs[a]	1,412	(10.1)	1,409	(10.1)	<0.01
RASIs					
Angiotensin receptor neprilysin inhibitors[a]	1,903	(13.6)	1,383	(9.9)	0.12
Other RASIs[a]	7,173	(51.2)	7,345	(52.4)	0.03
SGLT2 inhibitors[a]	2,209	(15.8)	1,835	(13.1)	0.08

[a]Parameters used to calculate propensity scores.

CCI, Charlson comorbidity index. ESAs, erythropoiesis-stimulating agents. HIF-PHIs, hypoxia-inducible factor prolyl hydroxylase inhibitors. IPTW, inverse probability of treatment weighting. NSAIDs, non-steroidal anti-inflammatory drugs. RASIs, renin-angiotensin system inhibitors. SD, standard deviation. SGLT2, sodium–glucose cotransporter-2. SMD, standardized mean difference.

Supplementary Table S7. All-cause mortality during on-treatment follow-up

	Patients	Deaths	Person-days	IPTW-adjusted HR [95%CI]
HIF-PHIs	13,357	430 (3.2%)	3,130,513	
ESAs	20,305	1,285 (6.3%)	3,325,425	0.38 [0.34–0.43]

On-treatment follow-up was censored at treatment discontinuation, treatment switching, dialysis initiation, loss to follow-up, or the end of the study period, as described in the Methods.

CI, confidence interval. ESAs, erythropoiesis-stimulating agents. HIF-PHIs, hypoxia-inducible factor prolyl hydroxylase inhibitors. HR, hazard ratio. IPTW, inverse probability of treatment weighting.

Supplementary Table S8. Baseline characteristics of HIF-PHIs and ESAs health checkup cohorts before and after IPTW

Characteristics	Crude			IPTW-adjusted		
	HIF-PHIs patients (n = 835)	ESAs patients (n = 1,113)	SMD	HIF-PHIs patients (weighted n = 800)	ESAs patients (weighted n = 1,114)	SMD
Male[a], n (%)	377 (45.1)	572 (51.4)	0.13	381 (47.6)	541 (48.6)	0.02
Age[a], years, average (SD)	85.3 (7.38)	83.7 (8.74)	0.19	84.6 (7.70)	84.4 (8.36)	0.02
Calendar year on index date[a], n (%)						
2020	12 (1.4)	141 (12.7)	0.45	36 (4.5)	87 (7.8)	0.14
2021	99 (11.9)	323 (29.0)	0.44	174 (21.8)	241 (21.6)	<0.01
2022	192 (23.0)	243 (21.8)	0.03	188 (23.5)	251 (22.5)	0.02
2023	268 (32.1)	256 (23.0)	0.21	224 (28.0)	298 (26.7)	0.03
2024	227 (27.2)	132 (11.9)	0.39	154 (19.3)	206 (18.5)	0.02
2025	37 (4.4)	18 (1.6)	0.17	24 (2.9)	32 (2.9)	0.01
Comorbidities, n (%)						
Anemia	825 (98.8)	1,096 (98.5)	0.03	791 (98.9)	1,099 (98.7)	0.02
Renal anemia	803 (96.2)	1,047 (94.1)	0.10	770 (96.2)	1,055 (94.7)	0.07
Atrial fibrillation[a]	315 (37.7)	377 (33.9)	0.08	291 (36.3)	401 (36.0)	0.01
Dyslipidemia[a]	532 (63.7)	712 (64.0)	0.01	518 (64.7)	720 (64.6)	<0.01
Hypertension	801 (95.9)	1,056 (94.9)	0.05	762 (95.3)	1,057 (94.9)	0.02
Hypotension	11 (1.3)	18 (1.6)	0.03	10 (1.2)	16 (1.5)	0.02
For CCI						
AIDS/HIV	0 (0)	0 (0)	NE	0 (0)	0 (0)	NE
Any malignancy	153 (18.3)	262 (23.5)	0.13	151 (18.9)	256 (23.0)	0.10

Characteristics	Crude			IPTW-adjusted		
	HIF-PHIs patients (n = 835)	ESAs patients (n = 1,113)	SMD	HIF-PHIs patients (weighted n = 800)	ESAs patients (weighted n = 1,114)	SMD
Cerebrovascular disease	276 (33.1)	399 (35.8)	0.06	276 (34.5)	389 (34.9)	0.01
Chronic pulmonary disease	245 (29.3)	374 (33.6)	0.09	242 (30.2)	365 (32.8)	0.05
Congestive heart failure	795 (95.2)	1,065 (95.7)	0.02	760 (95.0)	1,069 (96.0)	0.05
Dementia	146 (17.5)	176 (15.8)	0.05	133 (16.7)	174 (15.6)	0.03
Diabetes with chronic complication	111 (13.3)	180 (16.2)	0.08	108 (13.5)	168 (15.0)	0.05
Diabetes without chronic complication	65 (7.8)	84 (7.5)	0.01	59 (7.4)	84 (7.6)	0.01
Hemiplegia or paraplegia	7 (0.8)	16 (1.4)	0.06	7 (0.9)	13 (1.2)	0.03
Metastatic solid tumor	14 (1.7)	25 (2.2)	0.04	17 (2.1)	21 (1.9)	0.02
Mild liver disease	194 (23.2)	328 (29.5)	0.14	193 (24.1)	307 (27.5)	0.08
Moderate or severe liver disease	3 (0.4)	12 (1.1)	0.09	2 (0.3)	12 (1.1)	0.10
Myocardial infarction	80 (9.6)	77 (6.9)	0.10	80 (10.0)	77 (6.9)	0.11
Peptic ulcer disease	174 (20.8)	240 (21.6)	0.02	176 (22.0)	226 (20.3)	0.04
Peripheral vascular disease	176 (21.1)	295 (26.5)	0.13	181 (22.7)	276 (24.7)	0.05
Renal disease	820 (98.2)	1,063 (95.5)	0.16	787 (98.4)	1,069 (96.0)	0.15
Rheumatic disease	51 (6.1)	61 (5.5)	0.03	53 (6.6)	62 (5.6)	0.05
CCI (excluding age) [a], average (SD)	5.14 (1.86)	5.39 (1.98)	0.13	5.23 (1.92)	5.28 (1.91)	0.03
Concomitant medication, n (%)						
Beta blockers[a]	324 (38.8)	396 (35.6)	0.07	300 (37.5)	415 (37.2)	0.01
Calcium channel blockers	464 (55.6)	665 (59.7)	0.09	437 (54.7)	643 (57.7)	0.06
Diuretics						

Characteristics	Crude					IPTW-adjusted				
	HIF-PHIs patients (n = 835)		ESAs patients (n = 1,113)		SMD	HIF-PHIs patients (weighted n = 800)		ESAs patients (weighted n = 1,114)		SMD
Loop diuretics[a]	654	(78.3)	910	(81.8)	0.09	639	(79.9)	894	(80.3)	0.01
Potassium-sparing diuretics	341	(40.8)	380	(34.1)	0.14	312	(39.0)	397	(35.6)	0.07
Thiazide and thiazide-like diuretics	122	(14.6)	174	(15.6)	0.03	120	(15.0)	180	(16.2)	0.03
Vasopressin V2 receptor antagonists	104	(12.5)	146	(13.1)	0.02	95	(11.9)	151	(13.6)	0.05
Iron preparations	247	(29.6)	320	(28.8)	0.02	221	(27.6)	332	(29.8)	0.05
Non-topical NSAIDs	108	(12.9)	134	(12.0)	0.03	106	(13.3)	124	(11.1)	0.07
RASIs[a]	620	(74.3)	745	(66.9)	0.16	564	(70.6)	780	(70.0)	0.01
SGLT2 inhibitors[a]	133	(15.9)	112	(10.1)	0.18	101	(12.7)	138	(12.4)	0.01
Clinical parameters from health checkups										
BMI[a], kg/m^2, average (SD)	22.8	(3.74)	23.3	(3.66)	0.14	23.0	(3.78)	23.1	(3.57)	<0.01
eGFR[a], mL/min/1.73 m^2, average (SD)	34.3	(14.1)	33.6	(15.9)	0.05	33.7	(14.1)	33.8	(15.3)	<0.01
SBP[a], mmHg, average (SD)	132.6	(19.7)	133.2	(20.2)	0.03	132.6	(19.4)	132.7	(20.2)	0.01
Hb[a], g/dL, average (SD)	10.4	(1.36)	10.6	(1.54)	0.17	10.5	(1.42)	10.5	(1.5)	0.03

[a]Parameters used to calculate propensity scores.

BMI, body mass index. CCI, Charlson comorbidity index. eGFR, estimated glomerular filtration rate. ESAs, erythropoiesis-stimulating agents. Hb, hemoglobin. HIF-PHIs, hypoxia-inducible factor prolyl hydroxylase inhibitors. IPTW, inverse probability of treatment weighting. NE, not estimable. NSAID, non-steroidal anti-inflammatory drug. RASIs, renin-angiotensin system inhibitors. SBP, systolic blood pressure. SD, standard deviation. SGLT2, sodium–glucose cotransporter-2. SMD, standardized mean difference.

Supplementary Figure S1. Propensity score distributions in the HIF-PHIs and ESAs groups

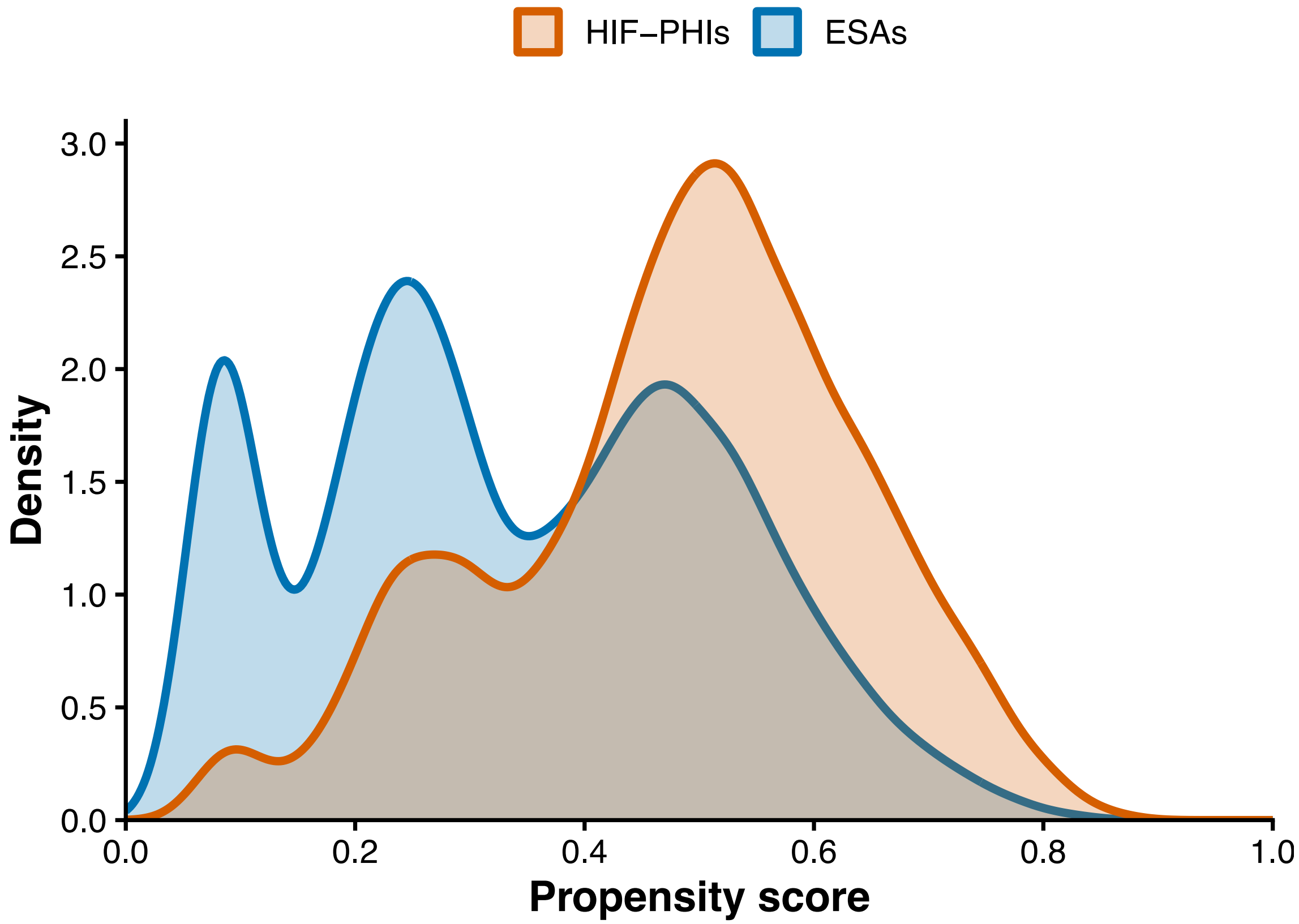

Density plots show the estimated propensity score distributions for the two treatment groups. Stabilized IPTW with 1st and 99th percentile weight trimming was subsequently applied in the outcome analyses.
